# A Neural Decision Signal during Internal Sampling from Working Memory in Humans

**DOI:** 10.1523/JNEUROSCI.1475-23.2024

**Published:** 2024-03-27

**Authors:** Freek van Ede, Anna C. Nobre

**Affiliations:** ^1^Institute for Brain and Behavior Amsterdam, Department of Experimental and Applied Psychology, Vrije Universiteit Amsterdam, 1081 BT, Amsterdam, The Netherlands; ^2^Oxford Centre for Human Brain Activity, Wellcome Centre for Integrative Neuroimaging, Department of Psychiatry, University of Oxford, Oxford OX3 7JX, United Kingdom; ^3^Department of Experimental Psychology, University of Oxford, Oxford OX2 6GG, United Kingdom; ^4^ Wu Tsai Institute and Department of Psychology, Yale University, New Haven, Connecticut 06510

**Keywords:** CPP, decision-making, EEG, ERP, selective attention, visual working memory

## Abstract

How humans transform sensory information into decisions that steer purposeful behavior is a central question in psychology and neuroscience that is traditionally investigated during the sampling of external environmental signals. The decision-making framework of gradual information sampling toward a decision has also been proposed to apply when sampling internal sensory evidence from working memory. However, neural evidence for this proposal remains scarce. Here we show (using scalp EEG in male and female human volunteers) that sampling internal visual representations from working memory elicits a scalp EEG potential associated with gradual evidence accumulation—the central parietal positivity. Consistent with an evolving decision process, we show how this signal (1) scales with the time participants require to reach a decision about the cued memory content and (2) is amplified when having to decide among multiple contents in working memory. These results bring the electrophysiology of decision-making into the domain of working memory and suggest that variability in memory-guided behavior may be driven (at least in part) by variations in the sampling of our inner mental contents.

## Significance Statement

A foundational question in the study of mind and brain is how we transform sensory information into decisions that steer adaptive behavior. This is traditionally investigated during the sampling of external environmental signals. Here, we demonstrate that a canonical EEG marker of decision-making from the human perception literature­—the central parietal positivity—also tracks gradual decision-making when selecting and accessing internally stored visual information from working memory. These findings bridge the literatures on decision-making and working memory and suggest that trial-to-trial variability in memory-guided behavior is driven, at least in part, by variations in the sampling of our inner mental contents.

## Introduction

How humans transform sensory information into decisions that steer purposeful behavior is a central question in psychology and neuroscience (Romo and Salinas, 2003; Gold and Shadlen, 2007; Cisek and Kalaska, 2010; Summerfield and De Lange, 2014; Gallivan et al., 2018; O’Connell et al., 2018). Traditionally this form of decision-making is studied using sensory stimulus streams containing noisy or mounting evidence, which is thought to be gradually accumulated and integrated until a decision is reached that guides behavior (Newsome et al., 1989; Shadlen and Newsome, 2001; Cisek et al., 2009; Donner et al., 2009; O’Connell et al., 2012).

Like perception, working memory also serves to link relevant (memorized) sensations to purposeful behavior (Fuster and Alexander, 1971; Baddeley, 1992; Rainer et al., 1999; de Vries et al., 2019; van Ede, 2020; Boettcher et al., 2021; van Ede and Nobre, 2023), and performance often involves deciding among multiple available representations (Chatham and Badre, 2015; Myers et al., 2017). Accordingly, it has been proposed—on the basis of both theoretical grounds (Shadlen and Shohamy, 2016) and behavioral observations (Ratcliff, 1978; Pearson et al., 2014; Shepherdson et al., 2018)—that the decision-making framework of gradual information sampling toward a decision may also apply when sampling internal sensory representations from working memory. Yet, neural evidence for this proposal has remained scarce (Shushruth et al., 2022), particularly in humans.

Here we aimed to address and bridge this gap in humans by taking advantage of the central parietal positivity (CPP)—a scalp EEG potential that has been shown to track gradual decision formation in humans (O’Connell et al., 2012; Kelly and O’Connell, 2013; Twomey et al., 2015, 2016; Herding et al., 2019; Brosnan et al., 2020; van den Brink et al., 2021). Previous CPP studies have required participants to make decisions based on external sensations (O’Connell et al., 2012; Kelly and O’Connell, 2013; Twomey et al., 2015, 2016; Herding et al., 2019; Brosnan et al., 2020; van den Brink et al., 2021). Instead, here we report a CPP-like signal when participants reached decisions based on internal visual evidence in working memory, in the absence of any external sensory evidence to sample. Moreover, complementary to the recent findings of delayed sampling of memorized random-dot-motion stimuli reported in Shushruth et al. (2022), we here report sampling of internal representations of stimuli that themselves did not require any temporal integration. Consistent with a gradual decision process, we show how this signal (1) scales with the time participants require to initiate their working-memory–guided report and (2) is larger when having to select (decide) among more than one representation in working memory.

## Materials and Methods

The current article presents the reanalyses of data from two prior studies (“E1” and “E2” for convenience), which each used a similar task setup. Complementary results from both prior studies were published previously [E1, van Ede et al. (2019); E2, Experiment 1 in Boettcher et al. (2021)]. These prior publications focused on distinct questions regarding the relation between visual working memory and action planning. Instead, here we report analyses prompted by contemporary studies on the CPP as a human EEG signature of perceptual decision-making (O’Connell et al., 2012; Kelly and O’Connell, 2013; Twomey et al., 2015, 2016; Brosnan et al., 2020; van den Brink et al., 2021). Our task designs and data proved to be ideally suited to address whether a CPP-like signature could also be established when human participants decide how to act based on internal representations in working memory.

### Participants

E1 contained data from 25 healthy human volunteers (14 female; age range, 19–36; 2 left-handed), as did E2 (18 female; age range, 18–35; all right-handed). Sample sizes of 25 were set a priori as described in van Ede et al. (2019) and Boettcher et al. (2021). Participants provided written informed consent before participation and received £15/hour for their participation. Experimental procedures were reviewed and approved by the Central University Research Ethics Committee of the University of Oxford.

### Task design and procedure

In both E1 and E2, participants performed a visual working-memory task with a delayed orientation–reproduction report from memory.

In E1 (van Ede et al., 2019), participants encoded two colored tilted bars (presented for 250 ms) into working memory. After a delay (drawn randomly between 2,000 and 2,500 ms) in which only the fixation cross remained on the screen, participants were cued to select one of the two memory items for report. The cue consisted of a color change of the central fixation cross. The task was to reproduce the orientation of the memory item that matched the color of the cue. At encoding, the two bars were positioned ∼5.7° visual angle to the left and right of fixation. The bars could be any of the four clearly distinguishable colors (blue, orange, green, purple) and could range in orientation between ±20 and ±70°. Bar location, color, and orientation varied independently across trials.

The orientation–reproduction report was performed by rotating a reporting dial (presented centrally, around fixation) to the memorized orientation of the cued item. The report was initiated by pressing either of the two keys on the keyboard (“\” or “/”) that were operated with the left and right index finger, respectively. The dial appeared on the screen once either key was pressed. Upon pressing the key, the dial would start in vertical position and would rotate leftward (key “\”) or rightward (key “/”) until the key is released, which terminated the response. The dial could be rotated maximally 90°. As a consequence, the tilt of the bar was directly linked to the hand required for responding: a left-tilted bar required a left-hand response, while a right-tilted bar required a right-hand response [see van Ede et al. (2019) for further details behind the rationale of this manipulation].

E2 (Boettcher et al., 2021) relied on the same basic task as E1 with two additions, of which the second is relevant to the current investigation. First, in E2, the memory delay was either 2 or 4 s [as motivated in Boettcher et al. (2021)]. This aspect was not of interest here, and we therefore collapsed trials with either delay duration. Second, and of direct interest, in E2 we included trials with a brief (250 ms) color precue that occurred 1 s before the encoding of the two memory items. Precues informed which item would be tested later. Accordingly, in these trials (comprising 80% of all trials in E2), participants needed to retain only a single item during the working-memory delay. Consequently, they no longer needed to select the relevant memory item before initiating their memory-guided report (which was still prompted by the second color cue at the end of the memory delay). Instead, in these trials, they could start deciding about the orientation and action as soon as the array appears and did not have to wait until the second color cue at the end of the memory delay. In the remaining 20% of the trials, the precue was neutral (gray), and participants were required to keep both items in working memory until the colored report cue indicated the relevant item to reproduce at the end of the delay (as in E1). This enabled us to compare the EEG response in the period between the reporting cue and the decision in trials in which the relevant memory item (1) had already been selected prior to the reporting cue or (2) still required to be selected after the report cue. Importantly, the memory report cue itself, as well as the ensuing reporting display and response requirements were identical between these two conditions.

In both E1 and E2, participants completed two consecutive EEG sessions of ∼50 min. The sessions were broken down into 10 blocks of ∼5 min. In E1, each block contained 60 trials, whereas in E2 each block contained 40 trials. This yielded 1,200 trials per participant in E1 and 800 in E2.

A critical feature of both E1 and E2 was that we probed (cued) internal working-memory content using a mere color change of the central fixation cross, without presenting any new visual element on the screen until response initiation (at which point the reporting dial appeared). For the current purposes, this is an important advantage over common working-memory tasks in which a perceptual “probe stimulus” is presented after the delay, to which a memory representation must be compared (such as in change-detection or delayed match-to-sample tasks). In such cases, the probe stimulus may itself trigger perceptual decision-making and yield a perceptually driven CPP. In contrast, in our tasks, the only perceptual element that cued the decision-making process was a color change of the central fixation cross. Crucially, while the color change was a perceptual element, our task was not to decide about the color but rather to sample the appropriate orientation information from working memory for the orientation–reproduction report. The color served merely as a medium to cue the correct internal representation. Moreover, because possible cue colors were highly distinguishable (blue, orange, green, purple), there were minimal demands on any “decision process” regarding perceived color itself.

Another important feature of our task was that once a key press was initiated, there was no return. The report terminated upon releasing the key, without the opportunity for further adjustment. Because of this, participants were required to deliberate carefully before committing to the initiation of their report.

### EEG acquisition and preprocessing

EEG acquisition and analyses followed the same procedures in E1 and E2. EEG data were collected using SynAmps amplifiers and Neuroscan software (Compumedics). Electrode placement followed the international 10-10 system, using a 61-electrode montage. Data were referenced to the left mastoid at acquisition. Measurements of vertical and horizontal EOG were included for off-line independent component analysis (ICA) correction. Data were digitized at 1,000 Hz.

EEG data were analyzed in MATLAB, using the FieldTrip toolbox (Oostenveld et al., 2011). Data were rereferenced to the average of both mastoids and downsampled to 250 Hz. ICA was performed to identify components associated with blinking or lateral eye movements, and bad components were removed from the data. After ICA correction, trials with exceptional variance were identified based on visual inspection (ft_rejectvisual with the “summary” method) and excluded. Noisy trials were identified without knowledge of the relevant conditions to which particular trials belonged. We additionally excluded trials in which the decision time (the time between cue onset and report onset) was below 200 or above 2,000 ms. In E1, on average 1,027 ± 25 trials (86 ± 2%) were retained for analysis. In E2, on average 700 ± 11 trials (87 ± 1%) were retained for analysis.

### CPP analyses

To identify the CPP, we zoomed in on an a priori defined electrode cluster centered on electrode Pz (“Pz,” “CPz,” “POz,” “P1,” “P2”), consistent with prior work investigating the P3 and CPP in the context of perceptual decision-making (Twomey et al., 2015).

To characterize the CPP, we aligned all trials to two moments: (1) the onset of the color cue at the end of the memory delay, which starts the decision-making process, and (2) the onset of the memory-guided reproduction report or “decision,” which signals the completion of the decision-making process. We focused our primary analyses on the decision-aligned data because the EEG signal in this alignment is less dominated by the sensory-driven ERP evoked by the abrupt color change of the cue. As such, the decision-aligned data provides a cleaner evaluation of the gradual buildup response to the decision—a key defining feature of the CPP (Twomey et al., 2015).

We baseline corrected the cue-locked data by subtracting the average EEG signal in the 250 ms window prior to cue onset. Likewise, we baseline corrected the decision-locked data by subtracting the average EEG signal in the 250 ms window following the response initiation. Baselining ensured that the trials were comparable with regard to the event to which we aligned the data, regardless of trial-to-trial variations in the time between cue onset and decision onset. We note that it is not trivial to decide on the appropriate baseline window within the context of our working-memory task. For example, before the cue (which in our case occurred at the end of a working-memory delay), there may be EEG differences related to variability in memory maintenance that may also have impact on decision times. This was the main reason that we decided to use the postresponse window for the primary decision-locked analyses of interest. At the same time, it is important to note that our estimates of the predecision CPP slopes—and their comparison across decision-time bins—do not hinge on the particular choice of baseline window because the slopes themselves are baseline independent. As an alternative to low-pass filtering, CPP time courses were smoothed with a Gaussian kernel (with a standard deviation of 30 ms).

To evaluate the relation between the CPP and decision times (defined as the time between cue onset and report initiation), we first sorted the data of each participant into nonoverlapping bins of decision times and calculated the average ERP in each bin. To leverage the large number of trials we had available in E1, we performed this analysis using 100 percentile bins per participant. After binning, we averaged the binned data across participants and visualized these as an ERP image (Jung et al., 2001), sorted by decision time (O’Connell et al., 2012). To facilitate additional visualization and quantification, we also performed the same analysis using four quartile decision-time bins per participant.

### Statistical evaluation

We used a cluster-based permutation approach (Maris and Oostenveld, 2007) to evaluate the baseline-corrected trial-averaged EEG potential time courses against zero (E1) and well between experimental conditions (E2). The cluster-permutation approach enabled us to evaluate statistically our data along the relevant time axes while circumventing the multiple-comparison problem.

To evaluate the link between the identified CPP and decision times, we compared the slope of the CPP across the four quartile decision-time bins. For the data from each quartile, we quantified the CPP slope as the linear regression coefficient between time and the trial-average EEG potential in the −500 to −50 ms window relative to response initiation. We quantified the gradient in this slope across the four quartile bins using a Pearson's correlation between bin number and slope coefficient. We obtained this correlation for each participant (first level) and quantified this at the group level (second level) using a one-sample *t* test against zero (with zero denoting the null hypothesis of no change in CPP slope across the four decision-time bins).

We restricted our statistical analyses to the EEG time courses extracted from the a priori defined electrode cluster. We additionally included topographical analyses to visualize the reported effects. Topographies were not subjected to additional inferential statistical testing. Instead, their visualization served primarily to increase transparency, to reinforce the suitability of our a priori electrode selection, and to demonstrate the “plausibility” of the reported findings (van Ede and Maris, 2016).

### Data availability

Data from both experiments have been made publicly available previously. Data from E1 are available through the Dryad Digital Repository (https://doi.org/10.5061/dryad.sk8rb66). Data from E2 are available through the Zenodo Repository (https://doi.org/10.5281/zenodo.4471943). Data from E2 in the current article are the data from “Experiment 1” in the latter data package that itself contains data from two experiments.

## Results

**Figure 1.**
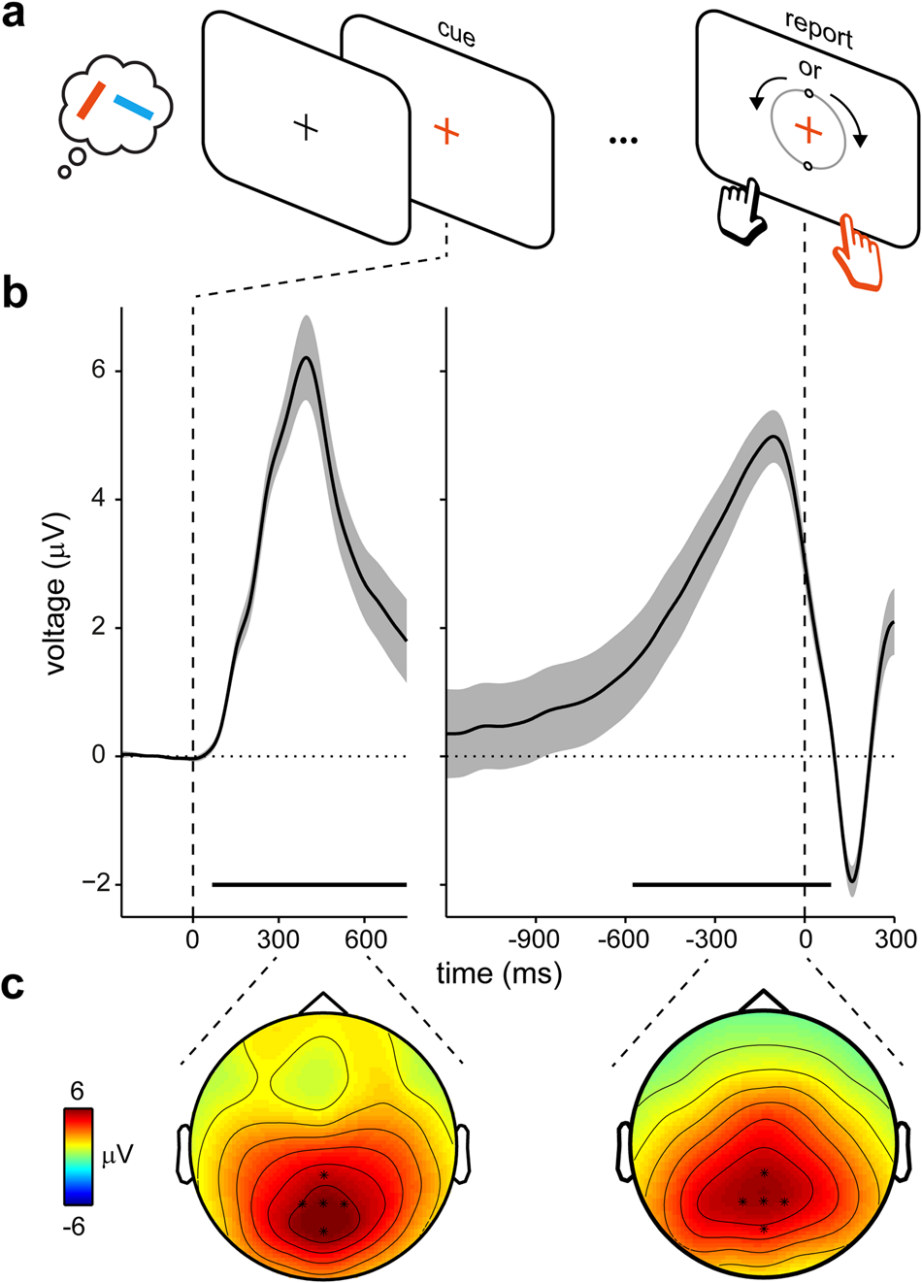
A CPP decision signal during internal sampling from visual working memory. ***a***, Visual working-memory task with a cued orientation–reproduction report [adapted from van Ede et al. (2019)]. Participants held two color-oriented bars in working memory until a color change of the central fixation cross cued them to report the orientation of the color-matching item from memory. The reporting dial appeared only upon report initiation (Figs. 2 and 3*a*,*b* for the distribution of decision times in this task). ***b***, Average EEG potentials in the selected central-parietal electrodes (indicated in ***c***) relative to memory cue onset (left) and report (decision) onset (right). Shading denotes ±SE, calculated across participants (*n* = 25). ***c***, Topographies associated with the EEG potentials in ***b***, averaged over the indicated time ranges (300–600 ms after cue and 300–0 ms before decision).

Participants held two tilted colored bars in visual working memory until a color change of the central fixation cross cued them to select and report the precise orientation of the color-matching bar from memory [Fig. 1*a*; see also van Ede et al. (2019) for a full task description and complementary results on this task]. Reproduction errors were on average 14.14 ± 0.84 (mean ± SEM) degrees, and it took participants on average 755.76 ± 52.29 ms to select the relevant item and to initiate the appropriate action.

### A CPP while sampling internal visual content from working memory

Analysis of EEG activity evoked by the memory cue revealed a pronounced positive potential (Fig. 1*b* left, cluster *p* < 0.0001) with a clear central-posterior topography (Fig. 1*c*, left). This potential is reminiscent of late positive potentials previously noted in working-memory tasks (Kok, 2001; Nieuwenhuis et al., 2005; Bledowski et al., 2006; Herding et al., 2019). Our task, however, had the distinguishing feature that we used, a symbolic cue (fixation-cross color change), to direct attention to internal working-memory content. This is different from traditional working-memory tasks in which decisions are made in the presence of a probe stimulus to which internal memory contents have to be compared (such as in change-detection and match-to-sample tasks). In our task, decisions about how to act did not concern the cue itself (apart from discerning its color) but rather the orientation of the internal memory content that was *indexed* by the cue.

Aligning these data to the moment at which participants decided to initiate their report revealed a clear gradual buildup of the potential toward this decision (Fig. 1, right; cluster *p* < 0.0001). This occurred despite no additional or changing sensory inputs in this decision period (the reporting display in Fig. 1*a* appeared only at report initiation). This buildup strongly resembles an accumulation-to-bound CPP signal similar to that observed in prior studies and attributed to a gradual decision signal (O’Connell et al., 2012; Kelly and O’Connell, 2013; Twomey et al., 2015, 2016; Herding et al., 2019; Brosnan et al., 2020; van den Brink et al., 2021).

These data thus reveal a CPP-like decision signal when sampling a cued internal representation from working memory in service of behavior. For convenience, we will also refer to this signal as the CPP, though we note that signal properties may differ from the more conventional CPP observed during sampling of external sensory evidence.

### The CPP has a central-parietal locus irrespective of the memorized item location and response hand

**Figure 2.**
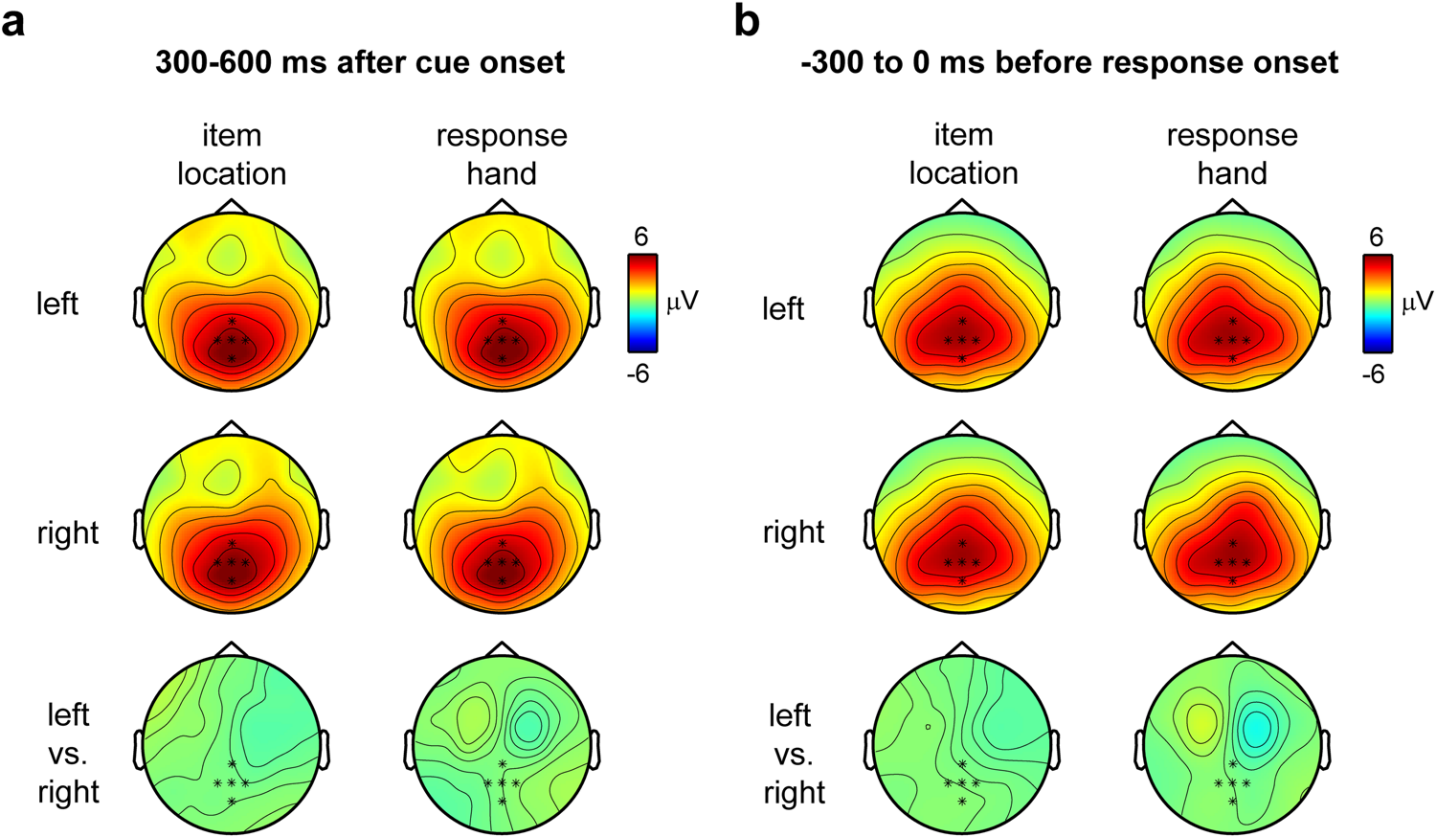
The CPP topography is invariant to the visual location and the manual action associated with the cued memory item. CPP topographies relative to cue (***a***) and decision (***b***) as a function of whether the cued memory content (item) was the left or right item at encoding and whether the reproduction report (action) required the left or the right hand.

Consistent with the aforementioned CPP literature, topographical analyses showed that the CPP always had a predominant contribution from an overlapping set of central-parietal electrodes, regardless of whether participants selected the left or right memory item or were required to make a left- or right-hand response (Fig. 2). At the same time, this does not mean there were no additional ERP components that lateralized according to item location and/or response hand (as was, in fact, the focus in our prior work; van Ede et al., 2019). Indeed, direct comparison of left- and right-hand response trials in the same time windows showed an additional ERP that lateralized according to response hand (Fig. 2 bottom row; here included for completeness). Unlike the more medial-posterior CPP locus, which is generalized across response hands, this lateralized ERP component had a more anterior locus, putatively overlaying left and right motor/premotor cortices. Further investigation into these additional lateralized components is beyond the scope of the current work, and we refer the reader to van Ede et al. (2019).

### The CPP during internal sampling scales systematically with decision times

**Figure 3.**
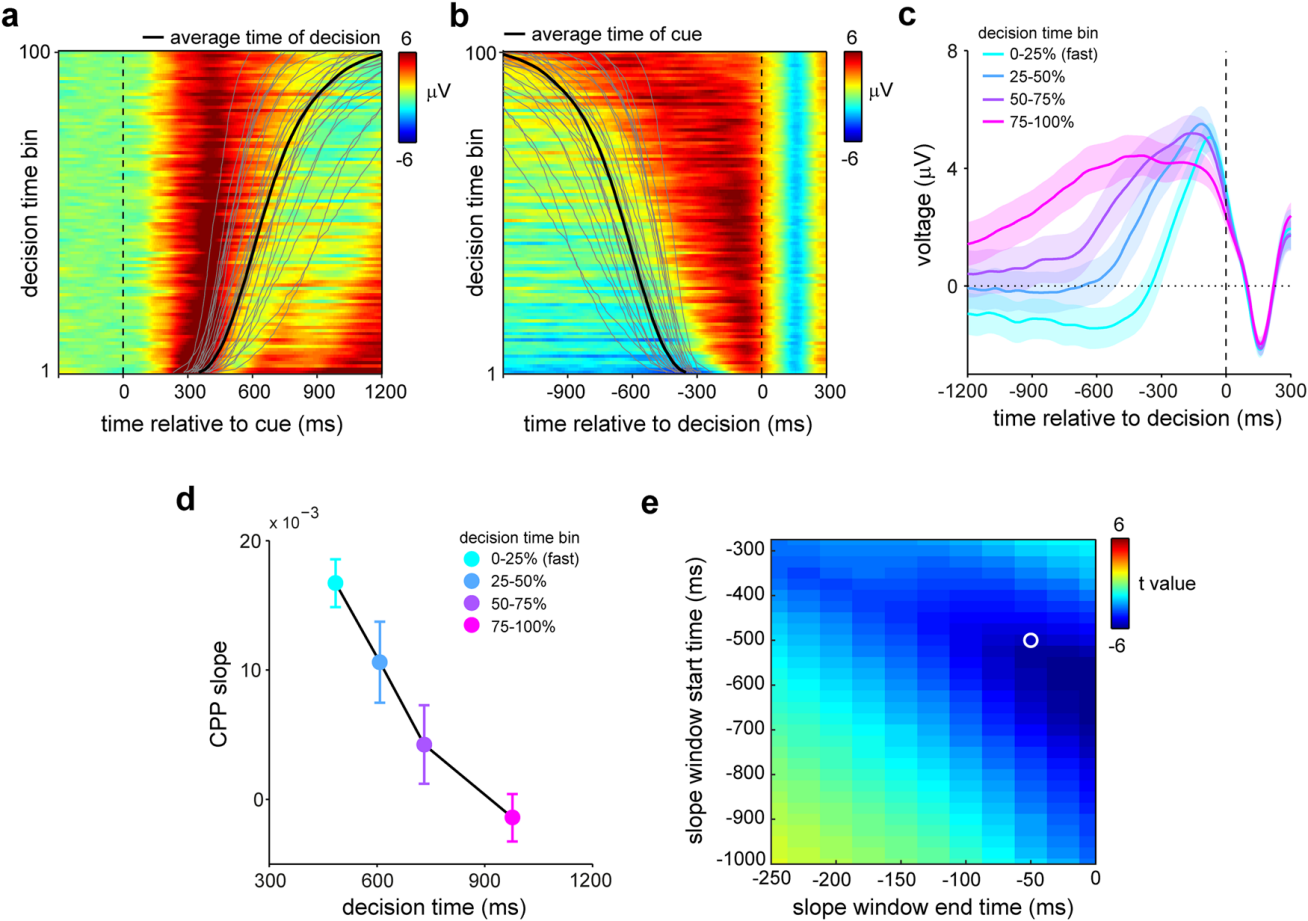
The CPP during internal sampling scales with decision time in an accumulation-to-bound fashion. ***a,b***, Average EEG potentials in the selected central-parietal electrodes locked to cue onset (panel ***a***) and report onset (panel ***b***) as a function of decision time. Data for each participant were binned into 100 percentile bins, and binned data were subsequently averaged across participants. The black line denotes the average decision time in each bin, while the gray lines show decision-time distributions of individual participants. ***c***, Overlay of average EEG potentials across quartile decision-time bins. ***d***, Slopes associated with the data in ***c***, calculated in the −500 to −50 ms predecision interval. Bins are positioned on the *x*-axis according to the average decision time in each bin. ***e***, *T* values of the correlation between CPP slope and decision-time bins as a function of the start and end of the time window used to estimate the slope. The white circle denotes the a priori chosen time window used for the data presented in panel ***d***. Shading in ***c*** and error bars in ***d*** denote ±SE, calculated across participants (*n* = 25).

To test whether the observed CPP reflected a decision-like process, we took advantage of the variability in decision times—the time from cue onset to report onset. We observed considerable variability in decision times (see individual decision-time distributions indicated by the gray lines in Fig. 3*a*,*b*), despite the relatively simple nature of our task. What may account for this variability in decision times when sampling from internal stores?

We found that the CPP varied systematically with decision time over the full range of values, ordered by decision-time percentiles in Figure 3, *a* and *b*. Because participants differed vastly in their decision-time distributions (indicated by the gray lines in Fig. 3*a*,*b*), the denoted average decision time per percentile bin (black line) is inevitably variable across participants. To understand how the data aligned with time of the decision, we therefore aligned all data to the moment of response onset (Fig. 3*b*). This revealed a strong correspondence between this signal and decision times; faster (slower) decisions were associated with a steeper (shallower) buildup of the CPP (Fig. 3*c*)—akin to what has been reported when deciding on external sensations (O’Connell et al., 2012; Kelly and O’Connell, 2013; Twomey et al., 2015; Brosnan et al., 2020; van den Brink et al., 2021). This relation between the CPP slope and decision time, quantified in Figure 3*d*, was highly robust across participants (average correlation between slope and decision-time bin, *r* = −0.725 ± 0.082 [M ± SE]; group-level evaluation, *t*_(24)_ = −8.858; *p* = 4.967 × 10^−9^; *d* = −1.772).

Note how this relation between the CPP slope and decision-time bins did not depend on our choice of baselining, as the estimates of the slope are baseline independent. Moreover, these findings were robust for a wide range of windows across which we calculated the slope. This is shown in Figure 3*e*, showing *t* values of the relation between decision-time bins and CPP slope as a function of the start and end times of the window used to calculate the slope. Our findings were thus not contingent on the specific a priori choice of slope window (indicated with the white circle in Fig. 3*e*).

Despite this clear link between the CPP and decision times, we did not observe reliable covariation between the CPP and response accuracy. This is likely owing to the relatively low variability in errors in our task (rendering low sensitivity to establish such a link), though it is also possible that the link between CPP and accuracy may be different in perception and working memory.

### The CPP is larger when having to select among alternative working-memory contents

**Figure 4.**
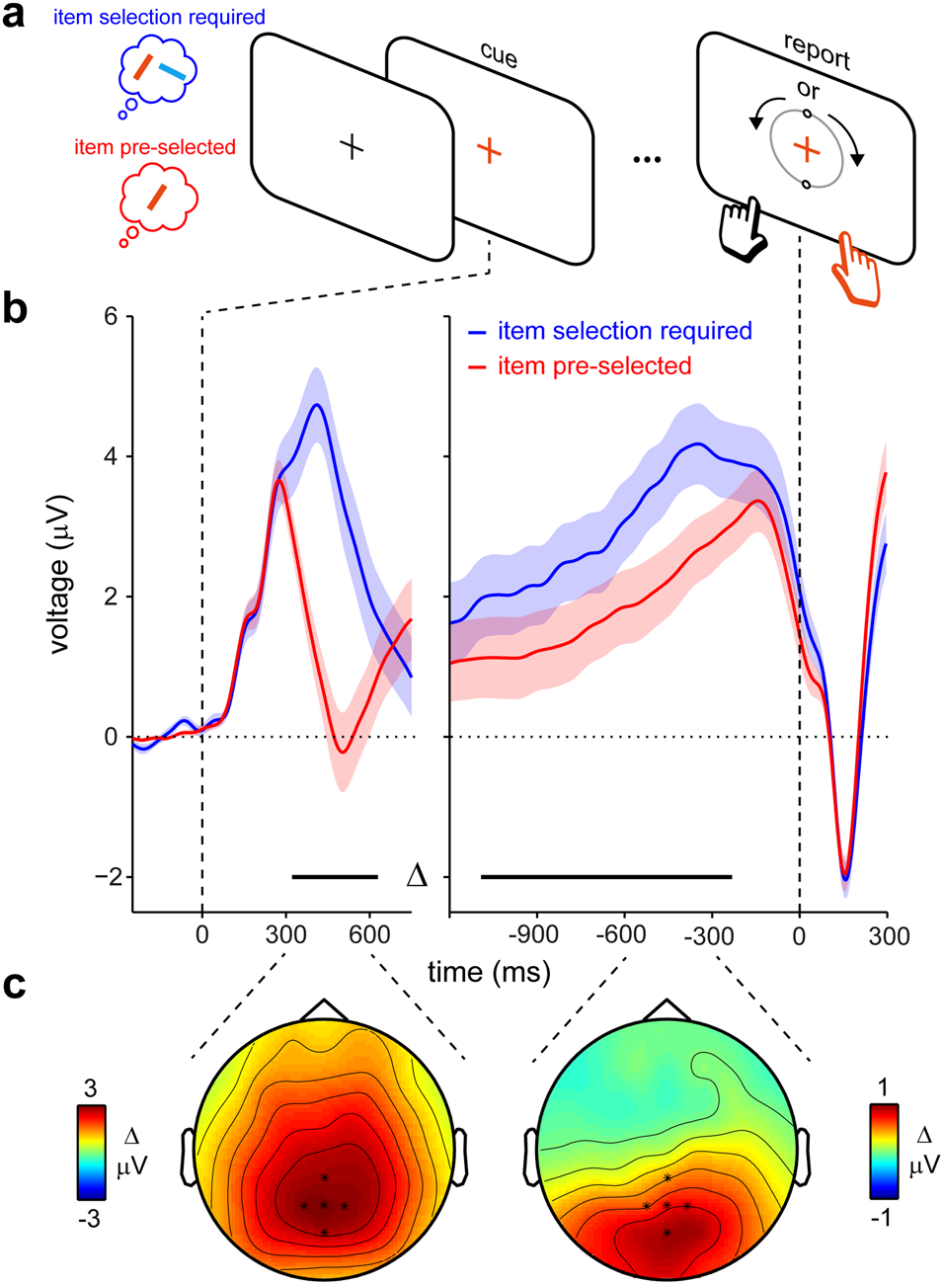
The CPP decision signal is larger when having to select among alternative working-memory contents. ***a***, Visual working-memory task with one (no item selection required) or two (item selection required) items held in working memory prior to the memory cue [adapted from Boettcher et al. (2021)]. The initial encoding displays (data not shown) always contained two items, of which one could be precued, reducing the effective memory load to 1 (red condition). ***b***, Average EEG potentials in the two conditions in the selected central-parietal electrodes relative to cue onset (left) and report (decision) onset (right). Horizontal lines denote significant differences between conditions (cluster-based permutation). Shading denotes ±SE, calculated across participants (*n* = 25). ***c***, EEG topographies associated with the condition differences in ***b***, averaged over the indicated time ranges (300–600 ms after cue and 600–300 ms before decision).

Finally, we asked whether this CPP signal during internal sampling depended on the requirement to select (decide) among multiple contents maintained in working memory. To this end, we reanalyzed the data from a similar experiment [of which complementary results are reported in Boettcher et al. (2021)] that differed in one key aspect. In this experiment, trials contained precues presented prior to memory encoding that could be either informative or neutral. In trials with a neutral precue, participants still had to select the relevant memory item at the end of the memory delay (as in Experiment 1; blue condition in Fig. 4). However, in trials with informative precues, participants could preselect the relevant memory item during initial memory encoding. Therefore, in these trials, only one item had to be retained in working memory, and there was no longer a need to decide among more than one memory representation following the report cue at the end of the memory delay (red condition in Fig. 4). In trials with a precue, in which the relevant item could already be selected at encoding, reproduction errors were on average 1.9° smaller (11.1 vs 13.0; *t*_(24)_ = −5.253; *p* = 2.1914 × 10^−5^; *d* = −1.051), and decision times were on average 253 ms faster (627.6 vs 880.9; *t*_(24)_ = −12.336; *p* = 7.0409 × 10^−12^; *d* = −2.467), compared with trials in which item selection was still required after the working-memory delay.

In this separate dataset, we replicated our main result of a CPP during internal sampling for working memory (Fig. 4*b*). Furthermore, these data revealed that this EEG potential was significantly larger when participants were required to select among competing memory representations in the decision period between cue and report initiation (blue vs red lines in Fig. 4*b*; topographies associated with these differences in Fig. 4*c*). This occurred both when aligning the EEG data to the report cue (Fig. 4*b*, left; cluster *p* < 0.0001) and to the decision (Fig. 4*b*, right; cluster *p* = 0.0026). We also observed that the CPP slope was steeper in the condition in which no item selection was required (*t*_(24) _= 2.583; *p* = 0.0163; *d* = 0.517), at least when using the slope window that we had used in Experiment 1. Though this difference appears more dependent on the choice of slope window as the effect reported in Experiment 1, this pattern is at least consistent with the lower decision times observed in this condition.

Note that the difference between conditions (in the magnitude of the CPP component) was observed despite the fact that the memory report cue and the ensuing reporting display and reporting requirements were identical. This difference between conditions that differed solely in effective memory load must therefore be attributed to the internal cognitive processes—the selection and sampling of the relevant internal representation—in the interval between the memory cue and the initiation of appropriate memory-driven behavior.

## Discussion

It has been posited that the decision-making framework of gradual evidence accumulation toward a decision may apply not only when sampling external sensations but also when sampling (“retrieving”) internal sensory representations in (working) memory (Ratcliff, 1978; Pearson et al., 2014; Shadlen and Shohamy, 2016). Yet, neural evidence for this proposal has remained scarce (Shushruth et al., 2022). Here we bridge this gap in humans. Building on the established link between the CPP and gradual decision formation during perceptually guided behavior (O’Connell et al., 2012; Kelly and O’Connell, 2013; Twomey et al., 2015, 2016; Herding et al., 2019; Brosnan et al., 2020; van den Brink et al., 2021), our CPP results suggest that similar neural decision processes may support working-memory–guided behavior.

Our results link variability in the speed of working-memory–guided behavior to variations in the sampling of internal memory contents—reflected in the CPP. This type of variability is not typically the focus in studies on visual working memory, which tend to focus on mechanisms of memory retention (during the delay) instead of utilization (after the delay). Yet, this type of variability is highly relevant from the perspective that working memory—just like perception—ultimately serves to translate (past) sensations into purposeful (future) behavior (Fuster and Alexander, 1971; Rainer et al., 1999; Myers et al., 2017; de Vries et al., 2019; van Ede, 2020; Boettcher et al., 2021; van Ede and Nobre, 2023). If we consider that internal memory representations are inevitably subject to noise (Rademaker et al., 2018; Panichello et al., 2019; Schneegans et al., 2020; Wolff et al., 2020) and may vary in their precision (van Den Berg et al., 2012), an internal evidence accumulation process may ensure optimal translation of noisy internal sensory representations into adaptive behavior.

The notion of gradual sampling of relevant sensory information toward a decision to guide behavior has a strong tradition in psychology and neuroscience (Newsome et al., 1989; Shadlen and Newsome, 2001; Romo and Salinas, 2003; Gold and Shadlen, 2007; Cisek et al., 2009; Donner et al., 2009; Cisek and Kalaska, 2010; O’Connell et al., 2012, 2018; Summerfield and De Lange, 2014; Gallivan et al., 2018). To date, however, this form of decision-making has been studied predominantly for decisions that regard currently available sensory information—even if it has been appreciated that memory processes often play a key role in reaching the best decision in such situations (Platt and Glimcher, 1999; Gould et al., 2012; Wimmer and Shohamy, 2012; Myers et al., 2015; Ramawat et al., 2023). Our data suggest that similar gradual decision formation may also apply in cases where the decision itself regards the visual identity of an internal working-memory representation. This brings together the study of working memory and decision-making—two topics that are often considered separately, despite their common focus on how the brain translates sensory information into purposeful behavior.

Our human data complement a recent study in nonhuman primates (NHPs) that reached a similar conclusion regarding sequential sampling from working memory (Shushruth et al., 2022). In their task, NHPs accumulated a random-dot-motion stimulus to decide where to look. Critically, the authors reported that the sampling of the stimulus would be protracted until the response mapping was provided after the motion stimulus—thus implying sampling from memory. While arriving at a similar conclusion, there are at least two notable differences between the current and this prior study. First, this prior study reported sequential sampling of a memory representation of a stimulus that itself has a sequential element (memorized dot motion). In contrast, in our study, the sensory memory representation itself did not require such temporal integration. As such, our current data extend the notion of sequential sampling from working memory to nonsequential memory content. Second, we report such sampling in humans. Our data further complement two other studies in human that reported a CPP to confidence judgments made after sensory evidence (Murphy et al., 2015) and to the comparison of an external tactile stimulus to a reference stimulus from tactile working memory (Herding et al., 2019).

Our data also build on studies of working memory (Kok, 2001; Nieuwenhuis et al., 2005; Bledowski et al., 2006) and long-term memory (Wilding, 2000; Finnigan, 2002; Rugg and Curran, 2007) that have previously implicated the P3 EEG potential (which is related to the CPP; Twomey et al., 2015) in the process of retrieval. Specifically, we uncover the ramping nature of this EEG potential (when aligned to report onset) and demonstrate this in the absence of any external stimulation contributing evidence to weigh into the decision. This is different from traditional working-memory tasks in which decisions are made in the presence of a probe stimulus which itself requires evaluation to support the comparison to internal memory contents (as in change-detection and match-to-sample tasks). This unique aspect of our task is also relevant when relating our CPP findings to prior studies reporting a gradual (decision-locked) CPP in tasks with isolated perceptual events (Twomey et al., 2015; van den Brink et al., 2021). In contrast to these studies, in which the decision regarded the perceptual stimulus itself, the decision in our task regarded memorized item orientation, a sensory feature exclusively contained in memory. Even so, it remains true that the CPP that we observed did not occur in the absence of any sensory input. Rather, it was prompted by a color change of the fixation cross that cued the relevant working-memory representation of another feature (orientation). While color perception itself could also be conceived of as a “decision process,” it is unlikely to account for the reported effects. Our decision times and CPP varied over a relatively wide range spanning several hundreds of milliseconds. It is unlikely that “deciding” what color you are seeing varies by this amount. Moreover, we found a larger CPP when having to decide among multiple memory contents (Fig. 4), despite identical color cues. Instead, such variation likely reflects variation in the sampling of internal working-memory representations.

We found that the CPP was larger when participants had to select among two visual items in working memory, compared with when the relevant memory item had already been preselected (contrary to prior reports of smaller probe-locked P3 responses with higher memory loads; Kok, 2001; Bledowski et al., 2006). There are at least two possible interpretations for this CPP amplification (which are not mutually exclusive). First, the process of selecting the correct memory item could itself be conceived of as a decision (that complements the decision about the required action given its orientation). Indeed, deciding among competing alternatives (in addition to the appropriate course of action) is a central component of decision-making (Rangel et al., 2008; Kolling et al., 2012; Wimmer and Shohamy, 2012; Hunt et al., 2018). Second, there may be differences in action-related decision-making. When the memory item that will become relevant is known in advance, planning for the appropriate course of action can start prior to the memory cue (Schneider et al., 2017; Boettcher et al., 2021; Nasrawi et al., 2023). This may alleviate elements of the decision-making process that would otherwise be possible only after the memory cue, such as deciding what hand to use for responding. It is worth noting how prior fMRI and MEG studies using retrocues have implicated various brain areas, some of which were suggested to be concerned with shifting attention while others with deciding what item to attend (Nobre et al., 2004; Bledowski et al., 2009; Wallis et al., 2015). The latter decision-related processes may be particularly relevant to the current findings.

In our setup, decisions guided actions. Therefore, it is possible that our CPP reflected some of the action-preparation consequences of decision-making processes that originated elsewhere. While our experiment was not designed to isolate pure decision-making from action preparation (cf. Urai and Pfeffer, 2014; Twomey et al., 2016; Sandhaeger et al., 2023), several relevant aspects of our data speak to this wider discussion. First, prior studies have elegantly shown how the CPP can be dissociated from effector-specific action planning, for example, showing up even when counting rather than directly responding to relevant targets (O’Connell et al., 2012). However, because we did not have such a condition, we cannot be certain that this previously established feature of the CPP also applies to our data (a reverse inference). At the same time, our data did provide relevant insight into this point: the CPP was most prominent in central-parietal electrodes—linked to the parietal cortex (Bledowski et al., 2006) by a prior EEG-fMRI study—regardless of whether the decision required a left- or a right-hand response. In addition, we found a larger CPP when participants had to decide among more than one content in memory, despite equivalent action-production demands (i.e., the same response dial, operated with the same keys on the keyboard) in both conditions (though we acknowledge that response readiness prior to the memory cue likely differed between conditions). Building on these initial observations, future studies can adopt experimental manipulations that specifically manipulate and disentangle decision-making versus action-preparation demands in the context of working memory. Having said that, we believe that a pure action-preparation account without any decision component may be insufficient to capture our data. We found that variability in the onset of memory-guided behavior was linked to variability in the CPP. For comparison, imagine an alternative scenario in which variability in the onset of memory-guided behavior was not due to gradual sampling but to variability in occasional “lapses of attention” after the memory cue. Such a scenario would yield a decision-locked EEG signal that would be invariant to the time leading up to the onset of the report.

Our inference regarding the decision process of sensory sampling from working memory hinges on the parallel between our findings and ample prior reports that have linked the CPP to decision-making (O’Connell et al., 2012; Kelly and O’Connell, 2013; Twomey et al., 2015, 2016; Herding et al., 2019; Brosnan et al., 2020; van den Brink et al., 2021). Because we did not directly manipulate decision-relevant variables (such as the strength of the evidence), our inference of a decision process ultimately relies on a reverse inference (Poldrack, 2006). Thus, having unveiled a CPP signal when accessing visual information from working memory—and a larger signal when also having to decide among competing memory items—our findings open the door for future studies to substantiate and further delineate the putative link between working memory and decision-making (cf. Ratcliff, 1978; Pearson et al., 2014; Shadlen and Shohamy, 2016; Shepherdson et al., 2018; Shushruth et al., 2022). Toward this endeavor, it will be vital to rule out alternative accounts, as outlined in Stine et al. (2020). Future studies should vary systematically the strength of the memory trace and apply drift–diffusion modeling (for which the current continuous–reproduction reports were not ideally suited). Our findings pave the way to such relevant future investigations.

Taken together, our results bring the electrophysiology of decision-making into the realm of working memory in humans and suggest that variability in memory-guided behavior may be accounted for (at least in part) by variations in the sampling of our inner mental contents.
